# Beneficial effects of intramyocardial mesenchymal stem cells and VEGF_165_ plasmid injection in rats with furazolidone induced dilated cardiomyopathy

**DOI:** 10.1111/jcmm.12558

**Published:** 2015-03-05

**Authors:** Qin Yu, Weiyi Fang, Ning Zhu, Xiaoqun Zheng, Rongmei Na, Baiting Liu, Lili Meng, Zhu Li, Qianxiao Li, Xiaofei Li

**Affiliations:** a Department of Cardiology, Affiliated Zhongshan Hospital of Dalian UniversityDalian, China; b Department of Cardiology, Shanghai Chest HospitalShanghai, China; c Department of Cardiology, The Second Affiliated Hospital of Dalian Medical UniversityDalian, China; d Department of Cardiology, Dalian Central HospitalDalian, China; e Zunyi Medical CollegeZunyi, China; f Department of Cardiology, Zhejiang Province Hospital of Integrated Traditional Chinese and Western MedicineHangzhou, China; g Linqu County People’s Procuraforate of Shandong ProvinceWeifang, China

**Keywords:** collagen, MSCs transplantation, hVEGF_165_ transplantation, dilated cardiomyopathy

## Abstract

To explore the impact of myocardial injection of mesenchymal stem cells (MSCs) and specific recombinant human VEGF_165_ (hVEGF_165_) plasmid on collagen remodelling in rats with furazolidone induced dilated cardiomyopathy (DCM). DCM was induced by furazolidone (0.3 mg/bodyweight (g)/day per gavage for 8 weeks). Rats were then divided into four groups: (*i*) PBS group (*n* = 18): rats received equal volume myocardial PBS injection; (*ii*) MSCs group (*n* = 17): 100 μl culture medium containing 10^5^ MSCs were injected into four sites of left ventricular free wall (25 μl per site); (*iii*) GENE group (*n* = 18): pCMVen-MLC2v-EGFP-VEGF_165_ plasmid [5 × 109 pfu (0.2 ml)] were injected into four sites of left ventricular free wall (0.05 ml per site)] and (*iv*) MSCs+GENE group (*n* = 17): rats received both myocardial MSCs and pCMVen-MLC2v-EGFP-VEGF_165_ plasmid injections. After 4 weeks, cardiac function was evaluated by echocardiography. Myocardial mRNA expressions of type I, type III collagen and transforming growth factor (TGF)-β1 were detected by RT-PCR. The protein expression of hVEGF_165_ was determined by Western blot. Myocardial protein expression of hVEGF_165_ was demonstrated in GENE and MSCs+GENE groups. Cardiac function was improved in MSCs, GENE and MSCs+GENE groups. Collagen volume fraction was significantly reduced and myocardial TGF-β1 mRNA expression significantly down-regulated in both GENE and MSCs+GENE groups, collagen type I/III ratio reduction was more significant in MSCs+GENE group than in MSCs or GENE group. Myocardial MSCs and hVEGF_165_ plasmid injection improves cardiac function possibly through down-regulating myocardial TGF-β1 expression and reducing the type I/III collagen ratio in this DCM rat model.

## Introduction

Dilated cardiomyopathy (DCM), a progressive disease of heart muscle, is a common cause of heart failure and the most frequent cause of heart transplantation 1,2. Cell therapy with mesenchymal stem cells (MSCs) represents a promising approach for alleviating cardiovascular injury and promoting tissue regeneration 3 and are under active investigation as a potential therapy for DCM 4,5. Previous studies showed that myocardial injection of MSCs improved cardiac function of rabbits with DCM *via* upregulating VEGF and its receptors 6 and myocardial injection of prokineticin receptor-1 (GPR73), a potent angiogenic factor, promoted cardiomyocyte survival and angiogenesis in infarcted mice 7.

Intracerebroventricular infusion of VEGF_165_ (5 μg/ml) decreased infarct volume and brain oedema after temporary middle cerebral artery occlusion without inducing a significant increase in cerebral blood flow suggesting that VEGF may have a direct neuroprotective effect in cerebral ischaemia 8. Westenbrink and colleagues showed that erythropoietin increased VEGF protein expression predominantly in cardiomyocytes and was associated with a 37% increase in capillary density and significantly improved cardiac performance in rats post-myocardial infarction while administration of the VEGF neutralizing antibodies abrogated the salutary effects of erythropoietin on cardiac microvascularization and function. VEGF neutralization also attenuated erythropoietin-induced proliferation of myocardial endothelial cells and reduced myocardial incorporation of endothelial progenitor cells in rats with alkaline phosphatase-labelled bone marrow cells, suggesting VEGF is crucial for improving cardiac function in heart failure animals 9. Formiga *et al*. demonstrated that VEGF_165_ administered as continuous release in border zone of a rat model of ischaemia-reperfusion promoted angiogenesis (small caliber caveolin-1 positive vessels), arteriogenesis (α-SMA, α-Smooth muscle actin positive vessels) and attenuated myocardial remodelling 10. Despite the promising experimental results indicating the beneficial effects of locally administrated MSCs and VEGF in various animal models, conflicting results were also reported and it was shown that overexpressing VEGF by Semliki Forest virus failed to induce cardiac angiogenesis and rather impaired systolic function in the mRen2 transgenic rat heart failure model 11. Taken together, most studies demonstrated beneficial effects of VEGF treatment in both cerebral and myocardial ischaemia model, although viral (especially the Semliki Forest virus) mediated overexpressing of VEGF might face viral-related negative effects.

Furazolodone could induce DCM in turkey poults 12 and rats 13. Previous studies showed that decreased energy reserve *via* the creatine kinase system contributed to reduced cardiac function in this DCM model 12, and morphometric analysis showed significant myocardial degeneration, interstitial fibrosis and mitochondrial swelling with fractured or dissolved cristae in furazolodone-fed rats 13. The effects of MSCs and VEGF in this model are not reported yet and we tested the hypothesis that combined myocardial MSCs and recombinant human VEGF_165_ plasmid injection might more efficiently improve cardiac function than MSCs or recombinant human VEGF_165_ plasmid injection alone in this rat model of furazolidone induced DCM 13.

## Materials and methods

### Reagents

Furazolidone (C8H7N3O5, MW 225.16, 99%) was purchased from Mongxin pharmaceutical of Chifeng Co, Ltd. (Chifeng city, Inner Mongolia Autonomous Region, China).

### Isolation and culture of bone marrow derived MSCs and human VEGF_165_ plasmid construction

Mesenchymal stem cells were obtained and the phenotype were identified by flow cytometry and immunofluorescence methods as described in detail by Karaoz *et al*. 14. Human VEGF_165_ plasmid was constructed as described previously 15.

### Animal model and study protocol

One-hundred and thirty-three Sprague–Dawley rats (17–32 g), provided by Shanghai Experimental Animal Center, were allowed free access to food and received furazolidone [43 mg/ml solution, 0.3 mg/bodyweight (g) by gavage] for 8 weeks. After 8 weeks, furazolidone was discontinued and survived rats (*n* = 70) underwent echocardiography examination (see below) and were anaesthetized with intramuscular ketamine hydrochloride injection (22 mg/kg), incubated and connected to a rodent ventilator, rat heart was exposed through a median sternotomy and randomly grouped and treated with following protocols: (*i*) PBS group (*n* = 18): rats received equal volume myocardial PBS injection into LV free wall; (*ii*) MSCs group (*n* = 18): rats received myocardial MSCs injection [(100 μl culture medium containing 10^5^ MSCs were injected into four sites of LV free wall (25 μl per site)]; (*iii*) GENE group (*n* = 17): rats received myocardial injection of pCMVen-MLC2v-EGFP-VEGF_165_ plasmid injection [5 × 10^9^ pfu (0.2 ml) at four sites of LV free wall (0.05 ml per site)] and (*iv*) MSCs plus GENE group (rats received both myocardial MSCs and pCMVen-MLC2v-EGFP-VEGF_165_ plasmid injections at four sites of LV free wall, *n* = 17). The chest was then closed with 3-0 silk sutures. All animals were treated in accordance with the Guide for the Care and Use of Laboratory Animals of the National Academy of Sciences (NIH publication no. 85-23, revised 1996). All animal study protocols were approved by the Institutional Animal Research and Ethics Committee of Dalian University.

### Echocardiography

Echocardiographic features were obtained under the recommendations of the American Society of Echocardiography 16. At baseline and at 8 weeks post-treatment with furazolidone and at 4 weeks after various treatments, survived rats were lightly anaesthetized with an intraperitoneal injection of ketamine (100 mg/kg). Left parasternal long-axis echocardiographic images of anaesthetized rats lying in a supine position were obtained with a Philips ultrasound system equipped with a 12.0 MHz transducer (HD11 XE; Philips Ultrasound, Bothell, WA, USA). To optimize the image, a transmission gel was used between the transducer and the animal’s chest. Animals were scanned from below at a depth of 2 cm with the focus optimized at 1 cm. All measurements were performed by the same observer based on the average of three consecutive cardiac cycles. LV dimensions were obtained from a parasternal long-axis view at the level of the papillary muscles. Left ventricular fractional shortening (LVFS) was calculated as (LVEDD-LVESD)/LVEDD×100, where LVEDD is LV end-diastolic diameter and LVESD is LV end-systolic diameter. Left ventricular ejection fraction (LVEF) was calculated according to the Teichholz formula 17.

### Tissue samples

After final echocardiographic examination, rats were killed under deep anaesthesia and the hearts were excised immediately, atria and right ventricle were separated from left ventricle, left ventricle was cut into two parts along long axis and one half of the left ventricle was fixed in 4% paraformaldehyde and stored at 4°C to undergo quantitative collagen content analysis with light microscopy studies. Heart sections (5 μm thick) were prepared with a cryostat at 2.5 mm interval. Cardiac fibrosis was evaluated by Masson staining and Sirius red staining through an automated image analyser (Image-Pro Plus 3.0; Media Cybernetics, Inc., Rockville, MD, USA). The collagen volume fraction (CVF = area of the collagen/area of field of vision × 100%) was measured. Ten separate areas of high power fields (100×) in each section were visualized under light microscope. Ten sections from each rat were observed and the results were averaged. The other half of the left ventricle was frozen in liquid nitrogen, and stored at −80°C for biochemistry studies (see below).

### Reverse transcription-PCR analyses

Myocardial collagen I, III and transforming growth factor (TGF)-β_1_ mRNA expression was detected as previously described 18,19. Total RNA was isolated from 100 mg LV tissue using the High Pure RNA Isolation Kit according to the manufacturer’s instructions (Roche Molecular Biochemicals, Indianapolis, IN, USA). Contaminated DNA was removed by treating the samples with RNAase-free DNAase Ι (Promega, Madison, WI, USA). Reverse transcriptase-PCR (RT-PCR) was performed with a ThermoScrip RT-PCR Kit following the manufacturer’s instruction Gibco Brl Life Technologies, Inc. (Indianapolis, IN, USA). The first-strand cDNA was synthesized by using oligonucleotide primers and M-MLV reverse transcriptase (Promega) before PCR amplification (35 cycles) using primers specific for rat collagen type I (5′-TGCCGTGACCTCAAGATGTG-3′ and 5′-CACAAGCGTGCTGTAGGTGA-3′), collagen type III (5′-CTG GAC CAA AAG GTG ATG CTG-3′ and 5′-TGC CAG GGA ATC CTC GAT GTC-3′), TGF-β_1_ (5′-GAA GCC ATC CGT GGC CAG AT-3′ and 5′-CCA GTG ACG TCA AAA GAC AG-3′) and GAPDH (5′-TCC GCC CCT TCC GCT GAT G-3′ and 5′-CAC GGA AGG CCA TGC CAG TGA-3′). All samples were subjected to RT-PCR for housekeeping gene GAPDH as a positive control and as an internal standard. Afterward, RT-PCR products were resolved on 1.5% agarose gels in 1× Tris-borate-EDTA buffer, visualized by ethidium bromide, photographed using a gel 1000 ultraviolet documentation system (Bio-Rad, Hercules, CA, USA) and analysed by densitometry.

### Western blot analysis

Myocardial protein expression of hVEGF_165_ was determined by Western blotting as described previously 20. Extracted protein lysates from LV tissue were separated on 7.5% SDS-PAGE and then transferred to Trans-Blot nitrocellulose membrane. Blots were then incubated with rabbit anti-human VEGF_165_ (1:400) antibody Calbiochem (San Diego, CA, USA) in PBS, then secondary goat anti-rabbit IgG (1:5000), or GAPDH (1:10,000) antibodies Pepro Tech Inc., (Rocky Hill, NJ, USA). The signal was developed by applying goat anti-rabbit IgG conjugated with horseradish peroxide Chemicon (CA, USA) and visualized with a diaminobenzidine system.

### Matrix metalloproteinase immunostaining analyses

Matrix metalloproteinase (MMP)-9 is a proximal biomarker for cardiac remodelling and angiogenesis. The expression of myocardial MMP-9 was examined with immunohistochemical analyses as previous reported 21. Hearts were fixed with formalin, embedded with paraffin and cut to obtain 5 μm sections. After incubation of sections for 10 min. with 0.3% H_2_O_2_, a serum-free protein block (DAKO, Carpinteria, CA, USA) was added for 10 min. Before adding MMP-9 primary antibody, the slides were treated with monohydrated citrate buffer (pH 6.0, 0.01 M) in a water bath for 10 min. at 100°C for the antigen retrieval. Sections were then incubated with the monoclonal antibodies against MMP-9 (1:100; Calbiochem®, San Diego, CA, USA) for 1 hour at room temperature. Anti-MMP-9 recognizes both latent and active form. Non-immune mouse serum was substituted for negative controls. After incubation for 10 min. with a biotinylated secondary antibody, AEC chromogen (DAKO) was used to develop the horseradish peroxidase-streptavidin complex.

### Cardiac angiogenic assessment

Blood vessels were highlighted by immunostaining for von Willebrand factor (vWF; 082; DAKO), visualized with diaminobenzidine. Vascular density was assessed by examining a single mid-papillary section (proximal to the injection site) from each heart and identifying all vWF-stained endothelial cell–lined structures in a total of eight high-power fields (magnification ×40) per region per heart.

### Statistical analysis

Data (mean ± SD) were analysed by one-way or two-way anova followed by Bonferroni’s post hoc comparisons with the SPSS (Statistical Product and Service Solutions), IBM (International Business Machines Corporation), (Armonk, NY, USA). A *P*-value <0.05 was considered as statistically significant.

## Results

### Identification of MSCs

**Figure 1:**
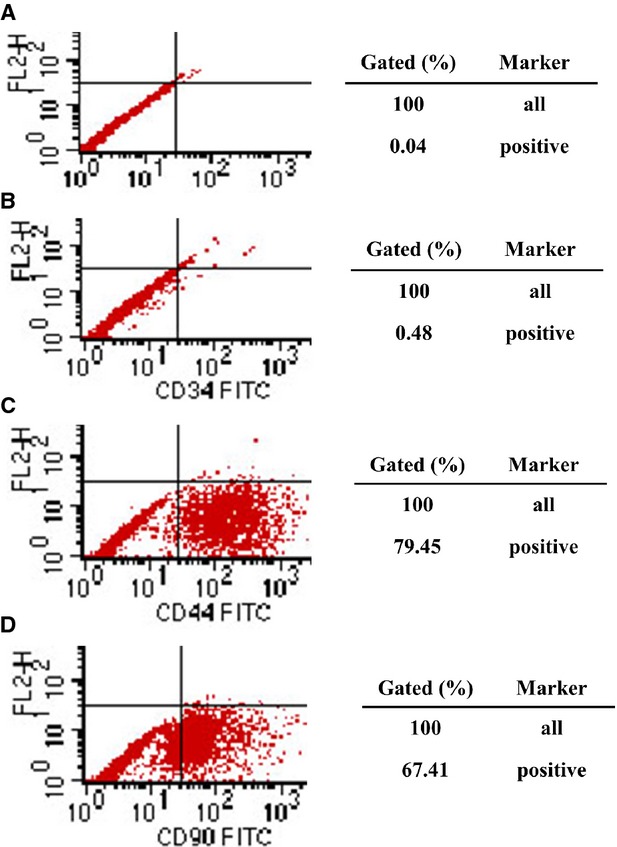
Representative results of identification of MSCs by fluorescence-activated cell sorting (FACS). (A) (negative control); (B) the positive rate of MSCs cells expressing CD34 was 0.48%; (C) expressing CD44 was 79.45%; (D) expressing CD90 was 67.41%.

Cultured MSCs were analysed and identified by fluorescence-activated cell sorting. Cells were incubated with fluorescein isothiocyanate–conjugated mouse monoclonal antibodies against rat CD44, CD34 Santa Cruz Biotechnology, Inc. (Santa Cruz, CA, USA), CD90 BD (Becton, Dickinson and Company., Franklin Lakes, NJ, U.S.A). Mesenchymal stem cells are known to express CD90, CD44 but not CD34. Our results showed that the positive rate of MSCs cells expressing CD34^+^ was 0.48% and expressing CD44^+^ was 79.45%, expressing CD90^+^ was 67.41% (Fig.1). Above expression patterns are consistent with MSCs 22.

### Animal survival rate and cardiac function at 8 weeks post-furazolidone and after another 4 weeks post-various therapies

**Figure 2:**
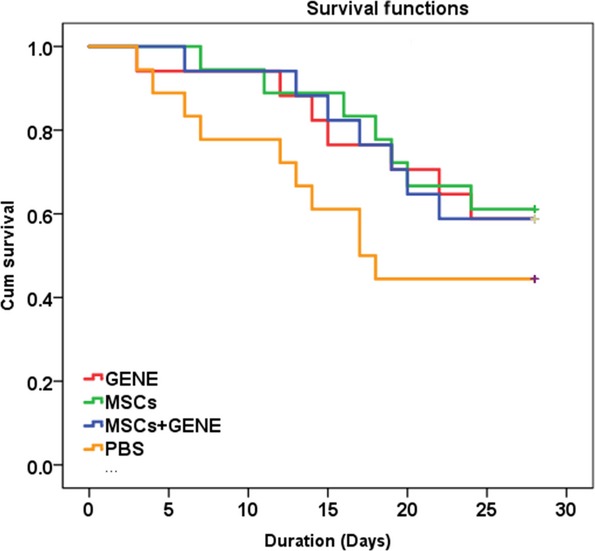
Kaplan–Meier survival curves. Survival was slightly improved in MSCs (green), GENE (red) and MSCs+GENE (dark blue) group compared to PBS (orange) group.

Seventy of 133 SD rats (52.6%) survived after 8 weeks treatment with furazolidone. After another 4 weeks, 8 of 18 rats (44.4%) in PBS group, 11 of 18 rats (61.1%) in MSCs group, 10 of 17 rats (58.8%) in GENE group and 11 of 17 rats (64.7%) in MSCs plus GENE group were alive (Fig.2). Only rats survived over the total 12 weeks study period were included in the final analysis.

### Echocardiography

LVEF and LVFS were comparable at baseline (Table1) and at 8 weeks post-furazolidone treatment (Table2). Four weeks after various treatments, LVEF and LVFS were significantly improved in MSCs and GENE groups and more significantly in MSCs plus GENE group compared to PBS group (Table2).

**Table 1:** Echocardiographic measurements at baseline

	Before therapy (*n* = 133)
LVEDD (mm)	4.8 ± 0.5
LVESD (mm)	1.5 ± 0.3
LVEF (%)	72 ± 5
LVFS (%)	53 ± 6

LVEDD: left ventricular end-diastolic dimension; LVESD: left ventricular end-systolic dimension; LVEF: left ventricular ejection fraction; LVFS: left ventricular fractional shortening.

**Table 2:** Echocardiographic measurements of DCM rats

	PBS (*n* = 8)	MSC (*n* = 11)	GENE (*n* = 10)	MSC+GENE (*n* = 11)
LVEDD (mm)				
8 weeks	5.8 ± 0.2	5.6 ± 0.5	5.6 ± 0.3	5.8 ± 0.3
12 weeks	5.6 ± 0.3‡	5.5 ± 0.4	5.6 ± 0.3	5.5 ± 0.3^‡^
LVESD (mm)				
8 weeks	4.1 ± 0.3	4.0 ± 0.4	4.0 ± 0.2	4.2 ± 0.3
12 weeks	3.9 ± 0.3	3.1 ± 0.3*,‡	3.6 ± 0.5‡	3.0 ± 0.4*,‡
LVEF (%)				
8 weeks	55 ± 5	54 ± 7	56 ± 6	54 ± 6
12 weeks	57 ± 4	74 ± 5*,‡	70 ± 6*,‡	78 ± 4*,†,‡
LVFS (%)				
8 weeks	29 ± 3	28 ± 5	30 ± 5	28 ± 4
12 weeks	32 ± 4‡	43 ± 5*,‡	36 ± 5‡	46 ± 6*,†,‡

Bonferroni adjusted

* *P* < 0.01 *versus* PSB

† *P* < 0.01 *versus* GENE

‡ *P* < 0.05 *versus* 8 weeks.

LVEDD: left ventricular end-diastolic dimension; LVESD: left ventricular end-systolic dimension; LVEF: left ventricular ejection fraction; LVFS: left ventricular fractional shortening.

### Collagen volume fraction and vWF

**Figure 3:**
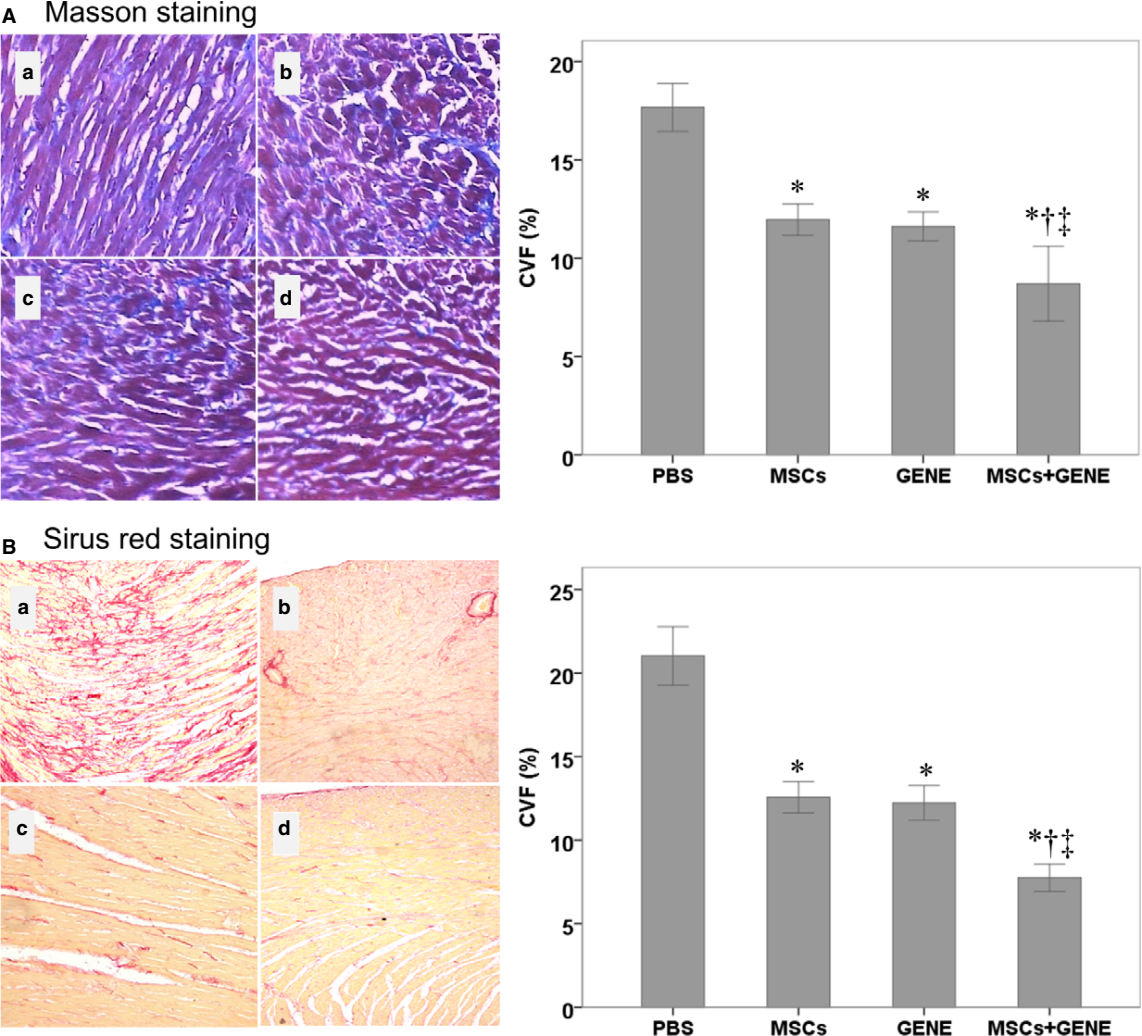
(A) Massion staining. (a) PBS; (b) MSCs; (c) GENE; (d) MSCs+GENE. Bar plots of collagen volume fraction in respective groups (right). (B) Sirius red staining. (a) PBS; (b) MSCs; (c) GENE; (d) MSCs+GENE. Bar plots of collagen volume fraction in respective groups (right). Note that CVF was significantly reduced in MSCs and GENE groups and further reduced in MSCs+GENE group compared to PBS group. Bonferroni adjusted *P < 0.01 *versus* PSB; †*P* < 0.01 *versus* GENE; ‡P < 0.05 *versus* 8 weeks.

**Figure 4:**
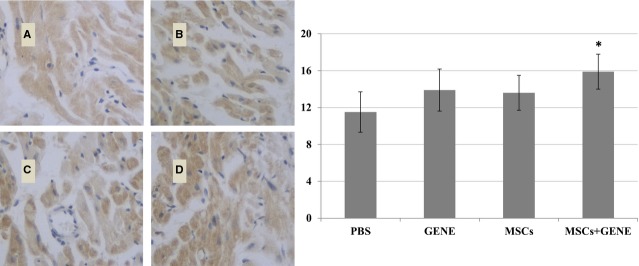
vWF staining. vWF expression tended to be higher in GENE (B) and MSCs (C) group and significantly higher in MSCs+GENE (D) group compared to PBS (A) group. *P < 0.05 *versus* PBS.

Collagen volume fraction was significantly reduced in MSCs and GENE group and further reduced in MSCs plus GENE group compared to PBS group at the end of study (Fig.3A: Massion staining and Fig.3B: Sirius red staining). von Willebrand factor staining showed a tendency for increased vWF expression in MSCs and GENE group and which was significantly up-regulated in MSCs+GENE group compared to PBS group (Fig.4).

### Myocardial protein expression of hVEGF_165_

**Figure 5:**
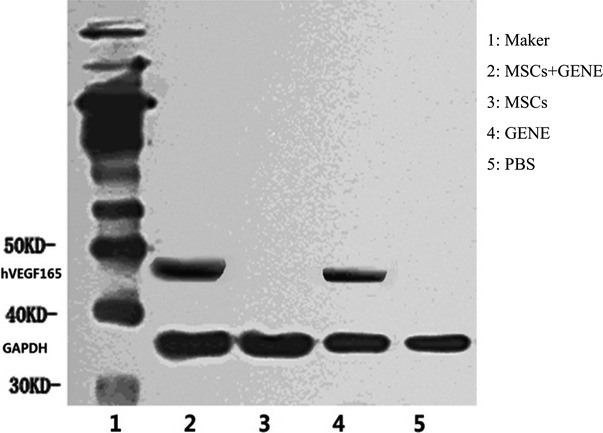
Representative hVEGF165 Western blot electrophoresis gel (1: Maker; 2: MSCs +GENE group; 3: MSCs group; 4: GENE group; 5: PBS group). Note that Western blot detected the presence of myocardial protein expression of hVEGF_165_ in GENE and MSCs+GENE group but not in PBS and MSCs groups.

Western blot detected the presence of myocardial protein expression of hVEGF165 in GENE and MSCs plus GENE group but not in PBS and MSCs groups (Fig.5).

### Myocardial mRNA expressions of TGF-β_1_, collagen I and III and MMP-9

**Figure 6:**
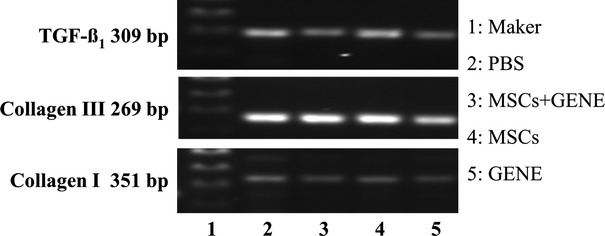
Representative RT-PCR electrophoresis gel (1: Maker; 2: PBS group; 3: MSCs+ GENE group; 4: MSCs group; 5: GENE group). Note that myocardial mRNA expression of TGF-β_1_ and collagen I were decreased in GENE and MSCs+GENE groups compared to PBS group, mRNA expression of collagen III was increased in MSCs group while decreased in GENE group. Collagen I/III ratio was reduced in MSCs and GENE groups and in MSCs+ GENE group.

**Figure 7:**
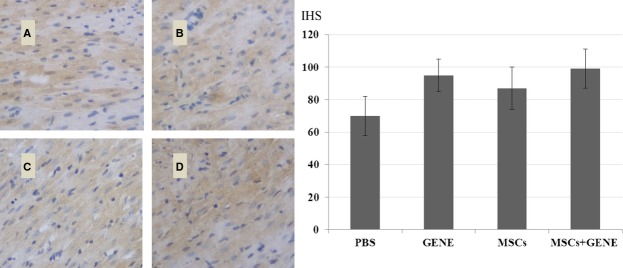
Myocardial MMP-9 expression. MMP-9 expression tended to be higher in GENE (B), MSCs (C) and MSCs+GENE (D) group compared to PBS (A) group.

The results of RT-PCR (Fig.6) showed that myocardial mRNA expressions of TGF-β_1_ and collagen I were down-regulated in GENE and MSCs+GENE group compared to PBS group, mRNA expression of collagen III was up-regulated in MSCs group while down-regulated in GENE group. Collagen I/III ratio was significantly reduced in MSCs and GENE group and further reduced in MSCs+GENE group. Myocardial MMP-9 expression also tended to be higher in MSCs and GENE groups compared to PBS group (Fig.7).

## Discussion

The major findings of this experimental study are as follows: (1) Both myocardial injection of hVEGF_165_ or MSCs as well as combined injection of MSCs and hVEGF_165_ improved myocardial function in furazolidone induced DCM rat model. (2) Observed beneficial effects by MSCs and hVEGF_165_ were linked with reduced CVF and down-regulated myocardial expression of TGF-β_1_, collagen I and collagen I/III ratio and higher expression of vWF and MMP-9. To our knowledge, that is the first report exploring the beneficial effects of myocardial MSCs and hVEGF_165_ injection in this furazolidone induced DCM rat model.

Our results obtained in this furazolidone induced DCM rat model are in line with previous findings by Nagaya and colleagues in a myocarditis and DCM rat model induced by immunizing the rat with porcine heart myosin and Freund’s complete adjuvant injection 4. In these two DCM models, MSCs transplantation resulted in reduced collagen deposit and improved cardiac function. Similarly, above mentioned beneficial effects post-MSCs transplantation were also demonstrated in this furazolidone induced DCM rat model and in a streptozotocin-induced diabetic cardiomyopathy rat model post myocardial phVEGF_165_ injection 23. Beyond these common findings, our results suggested the synergetic effects of combined myocardial injection of MSCs and hVEGF_165_ on cardiac function and myocardial collagen deposit.

Dilated cardiomyopathy is characterized by a loss of cardiomyocytes and an increase in fibroblasts (interstitial fibrosis) 24, thus, myocardial collagen deposit is a major player on the disease course of DCM. Despite numerous studies on the transplantation of MSCs in patient and animal models, insight into the mechanistic issues underlying the effect of MSC transplantation remains vague 25. Mesenchymal stem cells and VEGF induced myogenesis and angiogenesis as well as reduced myocardial collagen deposit are the major observations in both post-myocardial infarction or DCM models 26. As expected, our results demonstrated down-regulated myocardial mRNA expression of TGF-β_1_, collagen I in MSCs and GENE groups, and the down-regulation was more pronounced in MSCs+GENE group in this furazolidone induced DCM rat model. Taken together, these results suggest that reduction of myocardial collagen deposit might contribute to myocardial injection of MSCs and hVEGF_165_ induced beneficial effects on cardiac function in this furazolidone induced DCM rat model. Intensive studies suggest that VEGF is a major player mediating the beneficial effects of MSCs transplantation in ischaemic and heart failure animal models. Imanishi *et al*. showed that allogenic MSC transplantation has a therapeutic effect in acute myocardial infarction in rats, the donor MSCs disappear rapidly, but become a trigger of VEGF paracrine effect 27. Thus, it is reasonable to speculate simultaneous myocardial MSCs and hVEGF_165_ injection enhanced the VEGF paracrine effect in this model.

### Study limitations

It is to note that the study results evaluation in the present study was not performed in a blinded manner and which might induce potential bias on the reported results. Post-implantation survival of MSCs and expression of VEGF_165_ were not observed in the present study due to limited laboratory equipment. In a previous study, Jiang *et al*. demonstrated that intracoronary transfer of superparamagnetic iron oxide (SPIO) labelled heme oxygenase-1 (HO-1) overexpressed bone marrow stromal cells (BMSCs) in a porcine myocardial ischaemia/reperfusion model improved cardiac function, myocardial expression of VEGF was significantly higher in HO-1-BMSCs group than in Lacz-BMSCs group at 1 week post-transplantation. Signal voids induced by the SPIO were detected in the peri-infarction region in all BMSC groups at 1 week but not at 3 months post-transplantation and the extent of the hypointense signal was the highest in HO-1-BMSCs group. However, histological analysis showed that signal voids only represented cardiac macrophages that engulfed the SPIO-labelled BMSCs 28. Thus, paracrine effect of MSCs implantation might be a more important mechanism, despite the facts that most MSCs were engulfed by macrophages shortly after implantation. Excessive production of type I collagen by activated fibroblasts and myofibroblasts is the hallmark of fibroproliferative disorders. Vimentin is a member of type III intermediate filaments and is a marker of cells of 84 mesenchymal origin (*e.g*. fibroblasts and myofibroblasts) 29. It would be meaningful to observe vimentin expression in this DCM model and observe the impact of MSCs and VEGF on its myocardial expression, this was not done in the present study and future studies are warranted to highlight its role in the present study settings. Moreover, reverse-transcription-PCR in the era of real-time PCR was used in the present study, this limitation should be noted when interpreting the study results.

In conclusion, combined myocardial injection of MSCs and hVEGF_165_ plasmid efficiently improves cardiac function in this furazolidone induced DCM rat model, possibly through down-regulating TGF-β_1_ expression, reducing myocardial collagen deposit and collagen type I/III ratio. Future studies in large animal models are warranted to verify this finding and to prove if combined transplantation with both MSCs and hVEGF_165_ plasmid could be used as a feasible therapeutic strategy for the treatment of DCM.
